# BET protein inhibition regulates cytokine production and promotes neuroprotection after spinal cord injury

**DOI:** 10.1186/s12974-019-1511-7

**Published:** 2019-06-11

**Authors:** Judith Sánchez-Ventura, Jesús Amo-Aparicio, Xavier Navarro, Clara Penas

**Affiliations:** Institut of Neurosciences, Dept Cell Biology, Physiology and Immunology, Centro de Investigación Biomédica en Red sobre Enfermedades Neurodegenerativas (CIBERNED), Universitat Autonoma de Barcelona, Barcelona, Spain

**Keywords:** BET protein, Inflammation, Cytokine, Neuroprotection, Spinal cord injury

## Abstract

**Background:**

Spinal cord injury (SCI) usually causes a devastating lifelong disability for patients. After a traumatic lesion, disruption of the blood-spinal cord barrier induces the infiltration of macrophages into the lesion site and the activation of resident glial cells, which release cytokines and chemokines. These events result in a persistent inflammation, which has both detrimental and beneficial effects, but eventually limits functional recovery and contributes to the appearance of neuropathic pain. Bromodomain and extra-terminal domain (BET) proteins are epigenetic readers that regulate the expression of inflammatory genes by interacting with acetylated lysine residues. While BET inhibitors are a promising therapeutic strategy for cancer, little is known about their implication after SCI. Thus, the current study was aimed to investigate the anti-inflammatory role of BET inhibitors in this pathologic condition.

**Methods:**

We evaluated the effectiveness of the BET inhibitor JQ1 to modify macrophage reactivity in vitro and to modulate inflammation in a SCI mice model. We analyzed the effects of BET inhibition in pro-inflammatory and anti-inflammatory cytokine production in vitro and in vivo. We determined the effectiveness of BET inhibition in tissue sparing, inflammation, neuronal protection, and behavioral outcome after SCI.

**Results:**

We have found that the BET inhibitor JQ1 reduced the levels of pro-inflammatory mediators and increased the expression of anti-inflammatory cytokines. A prolonged treatment with JQ1 also decreased reactivity of microglia/macrophages, enhanced neuroprotection and functional recovery, and acutely reduced neuropathic pain after SCI.

**Conclusions:**

BET protein inhibition is an effective treatment to regulate cytokine production and promote neuroprotection after SCI. These novel results demonstrate for the first time that targeting BET proteins is an encouraging approach for SCI repair and a potential strategy to treat other inflammatory pathologies.

**Electronic supplementary material:**

The online version of this article (10.1186/s12974-019-1511-7) contains supplementary material, which is available to authorized users.

## Background

Spinal cord injury (SCI) commonly causes lifelong disability that reduces the quality of life and increases morbidity for the affected patients [1, 2]. Direct consequences of the injury are the loss of motor, sensory and autonomic functions, producing paralysis, anesthesia, and dysautonomia. Besides, neuropathic pain arises in many cases, further reducing the quality of life of SCI patients [3]. To date, there are no effective treatments available [2]. Thus, it is important to develop new therapeutic strategies to alleviate this harmful condition.

SCI courses with an inflammatory response that comprises mainly microglia and blood-derived macrophages [4, 5]. Both immune cells induce and magnify the immune response by secreting inflammatory molecules and influencing glial cells including astrocytes, oligodendrocytes, and microglia [6]. Thus, the reaction of the immune system induces a feed-forward response that produces a persistent pro-inflammatory microenvironment, which contributes to cell death and glial scar formation [2, 7]. Secreted pro-inflammatory cytokines (such as IL-1β, IL-6, TNF-α), as well as cytotoxic factors, including reactive oxygen and nitrogen species, contribute to enhance inflammation by recruiting inflammatory cells to clear debris at the injury area [8], but have the potential to cause toxicity and increase tissue damage [5, 6, 9, 10]. In addition, the persistent release of these pro-inflammatory modulators plays a critical role in the induction and maintenance of neuropathic pain [11]. In contrast, anti-inflammatory cytokines, such as IL-4, IL-10, and IL-13, help to suppress inflammation by reducing pro-inflammatory cytokine/chemokine production and contributing to wound healing and tissue remodeling [9, 10, 12].

Bromodomain and extra-terminal domain (BET) proteins belong to the reader family. BET proteins are characterized by the presence of two tandem bromodomains (BD1 and BD2) and an extra-terminal domain (ET), which are critical for their function [13, 14]. These proteins bind to acetylated lysine residues in both histone and non-histone proteins to recruit transcriptional complexes and thus regulate gene expression. As a result, genes related to proliferation and inflammation are expressed [13–15]. BET family includes BRD2, BRD3, BRD4, and BRDT, and among them, BRD4 seem to be the main responsible for pro-inflammatory gene expression by interacting with the acetylated lysine-310 at the p65 transactivation domain of NF-kB [14, 16]. Taking together, the inhibition of this epigenetic phenomenon is a promising target to control the inflammatory process.

Given the importance of BET proteins in inflammation, we hypothesized that these epigenetic mediators might be a therapeutic target after SCI. We used the BET inhibitor JQ1 and analyzed the effects after SCI. We observed that JQ1 produced a decrease of pro-inflammatory and an increase of anti-inflammatory cytokine expression acutely after SCI. We found that prolonged treatment with JQ1 reduced inflammatory response, increased neuroprotection, and enhanced functional outcome after SCI. Overall, the results of the present study indicate that BET proteins are a therapeutic target after SCI.

## Materials and methods

### Spinal cord injury

All experimental procedures were approved by the Universitat Autònoma de Barcelona Animal Experimentation Ethical Committee (CEEAH 1188R3-DMAH 6131) and followed the European Communities Council Directive 2010/63/EU. A total of 72 8-week-old female C57BL/6 mice were used for all the procedures and randomly divided into three groups depending on the experimental design. General anesthesia was induced by an intraperitoneal injection (i.p.) of a ketamine (90 mg/kg), xylazine (10 mg/kg), and saline mixture. After laminectomy, a moderate contusion was performed to the exposed spinal cord at T11 using the Infinite Horizon Impactor device (60 kDyn, tissue displacement between 450–600 μm). Animals received a i.p. dose of 30 mg/kg JQ1 diluted in 5% DMSO and 5% Tween-80 in saline 2 h post-operation and daily onwards until specified. Vehicle-treated animals received only the vehicle solution. Sham mice underwent the same surgery except the contusion injury.

### BMDM culture

Bone marrow-derived macrophages (BMDMs) were obtained from mouse femurs following a specific isolating protocol [17]. Briefly, femurs from 8-week-old C57BL/6 mice were cut in a sterile glass and the bone marrow cavity was flushed out with chilled PBS using 10-ml syringe with 25-G needle. Once the cells were isolated and centrifuged at 500×*g*, cells were cultured in DMEM/F-12 medium containing HEPES, 1% 200 mM l-glutamine, 10% fetal bovine serum, and 1% penicillin/streptomycin. In addition, to initiate macrophage differentiation, the medium was supplemented with 10 ng/ml of macrophage colony-stimulating factor (M-CSF; Stanley) for 9–10 days. During differentiation, the medium was replaced every 3 days to eliminate contaminating non-adherent cells. After the differentiation phase, adherent BMDMs were seeded at a density of 0.3 × 10^6^ cells/well in two different 6-well plates containing the medium above mentioned.

At days 9 and 10, DMSO (0.001%), JQ1 (1 μM), or LPS (100 ng/ml) alone or a combination of JQ1 and LPS was added to the cultures. First, cells were treated with 100 ng/ml of LPS to initiate the inflammatory response during 1 h. Then, either DMSO or 1 μM JQ1 was added for 2 h. Finally, cells were collected for RNA extraction.

### RNA isolation, RNA reverse transcription, and RT-qPCR

For in vitro analysis, RNA was extracted using the RNeasy Micro Kit (QIAGEN) according to the manufacturer’s recommendations. For in vivo analysis, adult mice were perfused with sterile saline 4 h (*n* = 24) or 4 days post-SCI (*n* = 14). Then, a 4-mm-long segment around the SCI epicenter was removed and snap-frozen. Tissue was homogenized with QIAzol lysis reagent (QIAGEN), and RNA was extracted using the RNeasy Mini Kit (QIAGEN) following the manufacturer’s guidelines.

RNA was quantified with a spectrophotometer (NanoDrop Technologies) and reverse-transcribed using an Applied Biosystems kit (Thermo Fisher Scientific). Then, the expression of target sequences was quantified by RT-qPCR using SYBR ® Green QPCR Master Mix (Agilent Technologies) and the corresponding primers (Table 1). Glyceraldehyde-3-phosphate dehydrogenase (GAPDH) was used as a housekeeping gene.

**Table 1:** List of primers used in RT-qPCR

Gene	F′	R′
ARG1	GTGAAGAACCCACGGTCTGT	CCAGAGATGCTTCCAACTGC
IL-1b	CTTCAAATCTCACAGCAGCACATC	CCACGGGAAAGACACAGGTAG
IL-4	CATGGGAAAACTCCATGCTT	TGGACTCATTCATGGTGCAG
IL-6	AACCACGGCCTTCCCTACTTCA	TCATTTCCACGATTTCCCAGAG
IL-10	GCTCCTAGAGCTGCGGACT	TCATTTCCGATAAGGCTTGG
IL-13	TCCAATTGCAATGCCATCTA	TGGGCTACTTCGATTTTGGT
CD206	ATTGTGGAGCAGATGGAAGG	ATTTGCATTGCCCAGTAAGG
CCL2	ATGGGTACCGTCACAACCTC	CCTGCTGCTGGTGATTCTCTT
CCL5	TGCCCACGTCAAGGAGTATTTCTA	TGGCGGTTCCTTCGAGTGACAA
CD68	CCAATTCAGGGTGGAAGAAA	ATGGGTACCGTCACAACCTC
CX3CR1	CTTTGGGGGCATATTCTTCA	ACGCCCAGACTAATGGTGAC
GAPDH	TGGCCTTCCGTGTTCCTAC	GAGTTGCTGTTGAAGTCG
GFAP	GGAGAGGGACAACTTTGCAC	CAGCCTCAGGTTGGTTTCAT
INOS	AATCTTGGAGCGAGTTGTGG	CAGGAAGTAGGTGAGGGCTTG
TNFa	AGGCACTCCCCCAAAAGATG	TCACCCCGAAGTTCAGTAGAC

### Cytokine protein expression

Adult female C57/Bl6 mice were perfused with sterile saline and a 5-mm-long segment of sham or the contused spinal cord was collected at 4 and 72 h after SCI (*n* = 3 per group and time point) and snap-frozen. The spinal cords were homogenized, and protein concentration was determined using the DC Protein Assay (Bio-Rad). Samples were concentrated to 4 mg/ml using MicroCon centrifugation filters (Millipore) to ensure equal amounts of protein. Cytokine protein levels were then analyzed using the Milliplex MAP Mouse Cytokine/Chemokine magnetic bead panel (Millipore) on a Luminex (Millipore) as per the manufacturers’ protocol.

### Functional assessment

Locomotion recovery was evaluated in an open-field test using the 9-point Basso Mouse Scale (BMS) at 3, 7, 10, 14, 21, and 28 days post-operation [18]. This scale ranges from 0, indicating complete paralysis, to 9, indicating normal movement of the hind limbs. The analysis was carried out by two researchers which strictly followed blinding procedures to determine the score for each animal. To evaluate neuropathic pain, mechanical and thermal algesimetry tests were performed. Mechanical nociception was measured once a week (at 7, 14, 21, and 28 days post-operation) by an electronic Randall-Selitto test [19]. Briefly, an increasing mechanical force was applied with a probe on the plantar surface of the hind paws until the animal produced retraction of the paw, indicating painful perception. Thermal algesimetry test [20] assesses the time that mice can endure heat irradiation. This test was performed at 28 days once the mice could sustain their weight on the hind paws.

### Histological analyses

Animals used for the functional assessment were sacrificed and perfused with 4% paraformaldehyde in 0.1 M phosphate buffer (PB) at day 28 (*n* = 16). An 8-mm length of the spinal cord containing the epicenter of the lesion was removed and cut into 20-μm-thick sagittal sections using a cryostat. A subset of sections was used to evaluate demyelination with Luxol Fast Blue staining (LFB; Sigma-Aldrich). Briefly, after dehydration, slides were placed in a 1 mg/mL LFB solution in 95% ethanol and 0.05% acetic acid at 37 °C overnight. Then, sections were washed in 95% ethanol and distilled water before being placed into a 0.05% Li_2_CO_3_ solution for 3 min at room temperature. Finally, after dehydration, sections were mounted in DPX mounting medium (Sigma-Aldrich). Myelin sparing of the three groups of animals was calculated by delineating the spared LFP-stained tissue area, and the total area, from images taken at the epicenter of the injury and every 100 μm rostral and caudal to the lesion site using ImageJ software. Then, the area of residual white matter was expressed as the percentage with respect to the total area of the spinal cord section.

For immunohistochemical staining, another subset of sections was rehydrated in PBS and blocked with 5% FBS in PBS 0.3Tx for 1 h at room temperature. The spinal cord sections were then incubated overnight at 4 °C with primary antibodies against glial fibrillary acidic protein (GFAP; for astrocytes; 1:1000; AB5804-Millipore), ionized calcium-binding adapter molecule 1 (Iba1; for macrophages/microglia; 1:500, ab5076-Abcam), and NeuN (for neurons; 1:75; MAB377x-Millipore, conjugated with Alexa 488). Nuclei were labeled with DAPI staining (1:1000; D9564-10MG-Sigma). The detection was made with appropriate secondary antibodies conjugated with Alexa Fluor 594 (A11058 for Goat and A21207 for Rabbit; Invitrogen). Finally, after several washes in PBST-Tween 0.1%, PBS and PB, a coverslip was applied in Fluoromount-G mounting medium (Southern Biotech). Microphotographs were taken at × 20, and then, immunoreactivity was analyzed by measuring the integrated density of a region of interest (ROI) after defining a threshold for background correction. The ROI had an area of 0.05 mm^2^ and was placed on the gray matter and on the white matter. Both markers were measured from 7 spinal cord sections (separated 200 μm) of each animal. Finally, neuronal survival was assessed by counting the number of neurons located rostrally and caudally from the epicenter in both ventral horns. Measurements were performed with ImageJ software.

### Data analysis

Data are expressed as the mean ± standard error of the mean (SEM), and results were analyzed using GraphPad Prism 7 software. For statistical analysis of cytokine/chemokine RNA expression, one-way analysis of variance (ANOVA) followed by Tukey’s post hoc test was performed. For cytokine protein expression, unpaired *t* test was performed. Functional test results were analyzed by two-way ANOVA followed by Sidak correction for multiple comparisons. Finally, histological quantification results, including tissue sparing and immunohistochemistry, were compared by two-way ANOVA followed by post hoc Tukey’s multiple comparison test. All differences were considered statistically significant when *p* < 0.05.

## Results

### BET inhibition reduces pro-inflammatory and enhances anti-inflammatory cytokine production

**Fig. 1:**
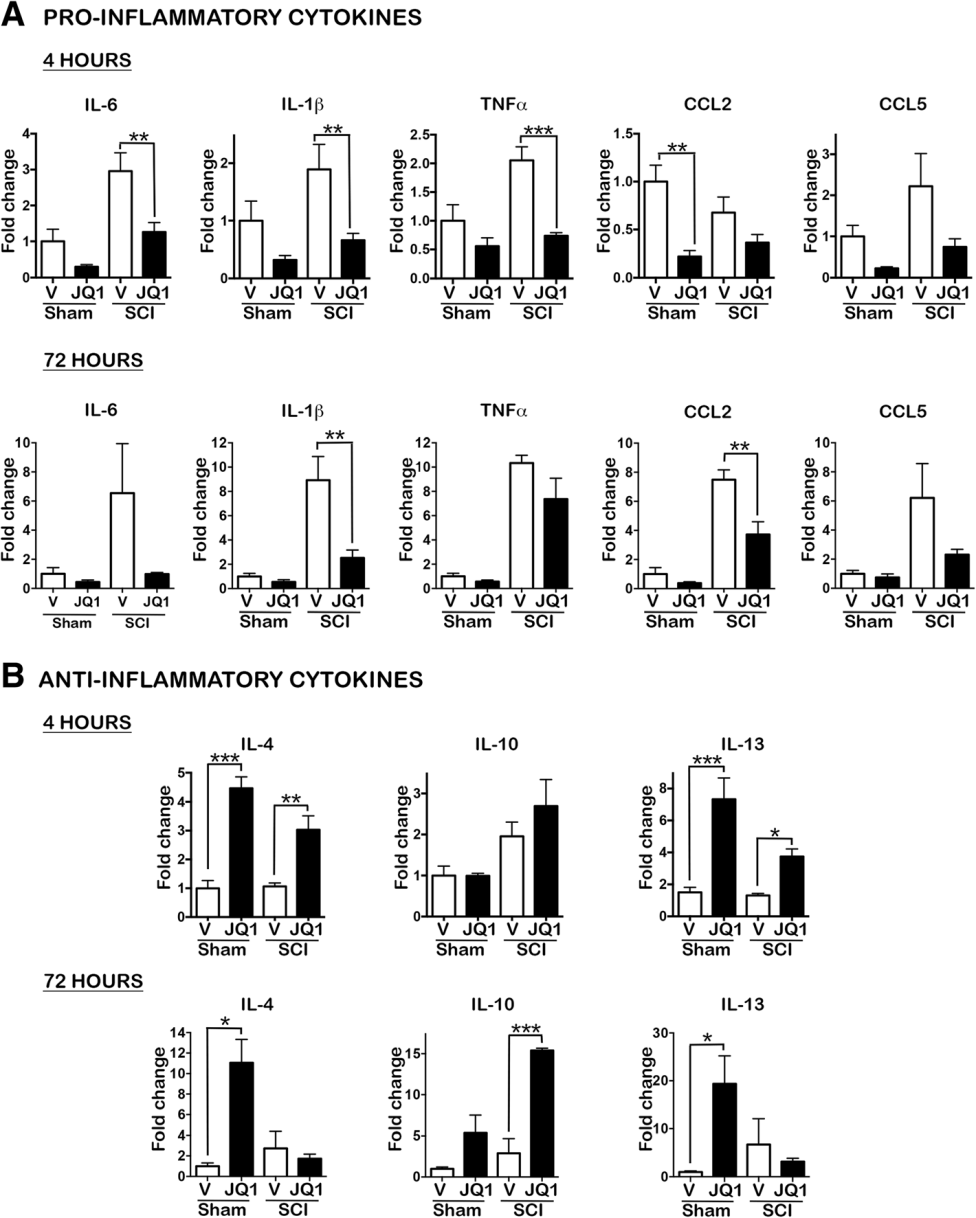
BET inhibitor JQ1 reduces expression of pro-inflammatory and increases expression of anti-inflammatory cytokines and chemokines acutely after SCI. Real-time PCR quantification of **a** pro-inflammatory cytokines and chemokines at 4 and 72 h after SCI and **b** anti-inflammatory cytokines at 4 and 72 h after injury, normalized to the GAPDH levels. *N* = 4–5 mice per group and time. *p < 0.05, **p < 0.01, ***p < 0.005, ****p < 0.001 by one-way ANOVA followed by Tukey post hoc test. Data are represented as mean ± SEM fold changes of gene expression

After SCI, a large number of pro-inflammatory markers become upregulated. To investigate the efficacy of BET inhibition to modulate the secondary injury response, we monitored the expression of inflammation-related genes after SCI or in sham mice treated with the BET inhibitor JQ1 or with vehicle. As shown in Fig. 1a, at 4 h post-lesion, the expression of the pro-inflammatory cytokines IL-6, IL-1β, and TNF-α was significantly reduced in injured mice treated with JQ1 compared to vehicle-treated mice. From these cytokines, IL-1β was maintained downregulated at 72 h post-lesion. We also detected a general decrease in chemokine levels after JQ1 treatment but only significantly for CCL2 at 72 h in injured mice treated with JQ1 compared to vehicle-treated mice and for CCL2 at 4 h and CX3CR1 at 72 h post-lesion (Additional file 1: Figure S1B) in sham-treated mice compared to vehicle-treated animals. These results validate previous studies [21].

**Fig. 2:**
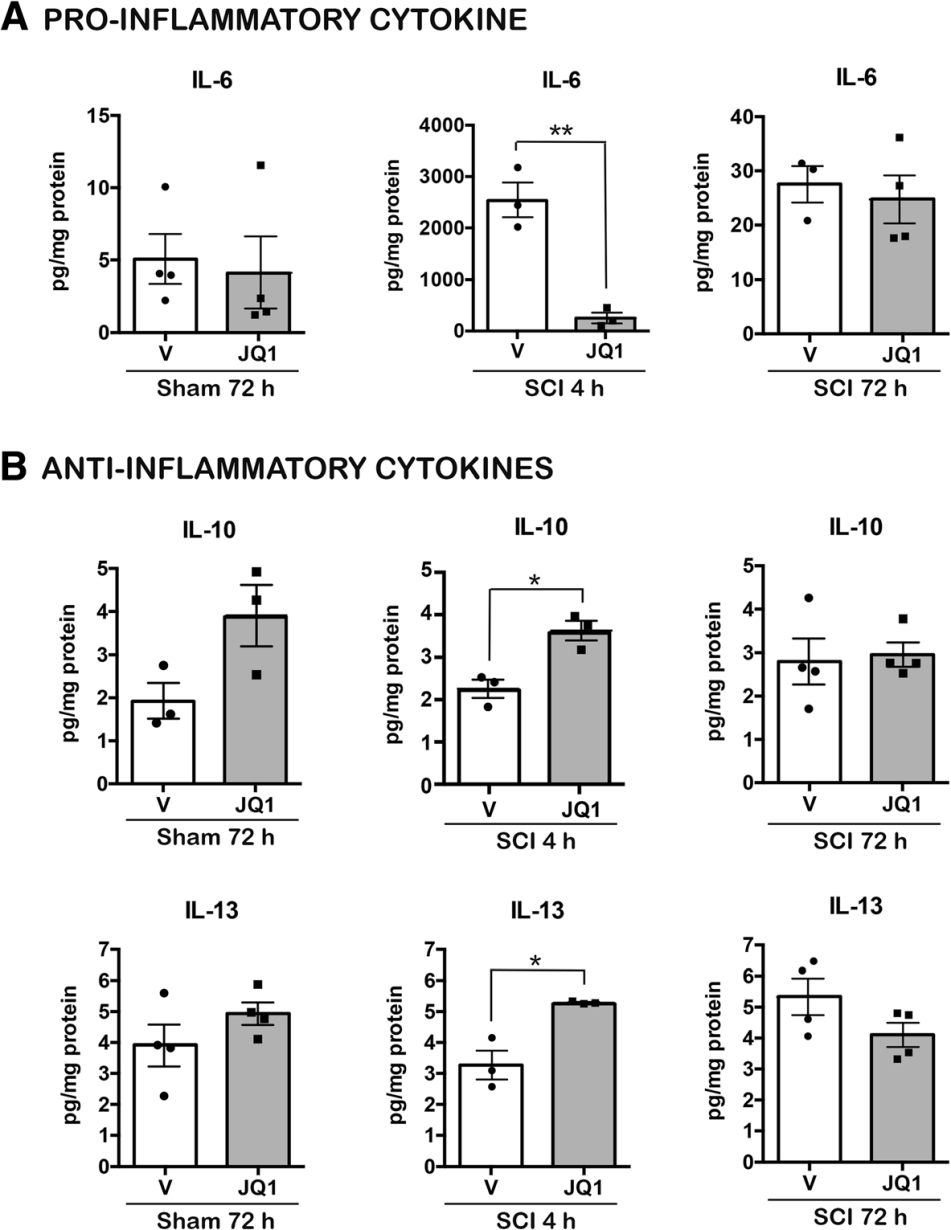
BET inhibitor JQ1 decreases pro-inflammatory and increases anti-inflammatory cytokine protein expression after SCI. The protein levels of the pro-inflammatory cytokine IL-6 (**a**) and the anti-inflammatory cytokines IL-10 and IL-13 (**b**) were quantified via Luminex analysis at 4 and 72 h after operation. *N* = 3–4 mice per group and time point. *p < 0.05, **p < 0.01, unpaired *t* test

In addition, our results revealed that the anti-inflammatory effect of JQ1 resides not only on reducing pro-inflammatory markers but also to upregulate anti-inflammatory cytokines. JQ1 increased IL-4 and IL-13 mRNA expression at 4 h post-injury and IL-10 levels at 72 h after SCI (Fig. 1b). To note, JQ1 increased IL-4 and IL-13 also in sham-treated animals (Fig. 1b). To ascertain if the changes observed on cytokine mRNA levels were traduced on protein levels, we analyzed IL-4, IL-10, IL-13, and IL-6 protein levels after SCI. IL-4 levels were undetectable in most of the samples because the detection levels of the assay were too high for the biological levels of the protein (data not shown). However, we corroborated the increase of IL-10 and IL-13 in sham and injured mice treated with JQ1 compared to vehicle-treated mice (Fig. 2). We also corroborated a decrease of IL-6 protein expression at 4 h post-injury in JQ1-treated animals compared to vehicle-treated mice.

In addition, other inflammatory markers related to macrophage infiltration and microglial activation were studied. Considering macrophages and microglia, JQ1 treatment after SCI greatly reduced the pro-inflammatory macrophage marker INOS at 72 h post-lesion (Additional file 1: Figure S1), although it did not affect the anti-inflammatory macrophage markers ARG1 and CD206. Thus, apparently, BET inhibition at the dosage used did not modify the balance of M1/M2 macrophage phenotype. However, a higher number of M1/M2 markers and fluorescence-activated cell sorting (FACS) should be performed to confirm these results. Besides, astrocyte reactivity determined by GFAP expression was not affected by JQ1 administration (Additional file 1: Figure S1B).

Finally, we investigated whether the effects observed after BET inhibition in SCI could be produced by affecting macrophage reactivity. To simulate the performed in vivo studies, and as a difference with previous similar studies where macrophages where pre-treated with JQ1 [15, 21], primary cultures of BMDMs received a stimulation with LPS during 1 h and followed or not by JQ1 treatment during 2 h. Treatment with JQ1 resulted in a reduction of the pro-inflammatory modulators IL-6 and INOS after LPS stimulation (Additional file 2: Figure S2). Regarding the anti-inflammatory cytokines IL-4, IL-10, and IL-13, we found that administration of JQ1 upregulated their transcription. For IL-10 and IL-13, the upregulation after JQ1 treatment was found with and without previous LPS stimulation, and for IL-4, only without LPS stimulation (Additional file 2: Figure S2).

Overall, while JQ1 suppressed the expression of key pro-inflammatory genes, it also produced an early expression of anti-inflammatory cytokines after SCI. The changes observed in mRNA levels were concomitant with changes on protein expression after SCI.

### BET inhibition reduces microglia/macrophage reactivity after SCI

**Fig. 3:**
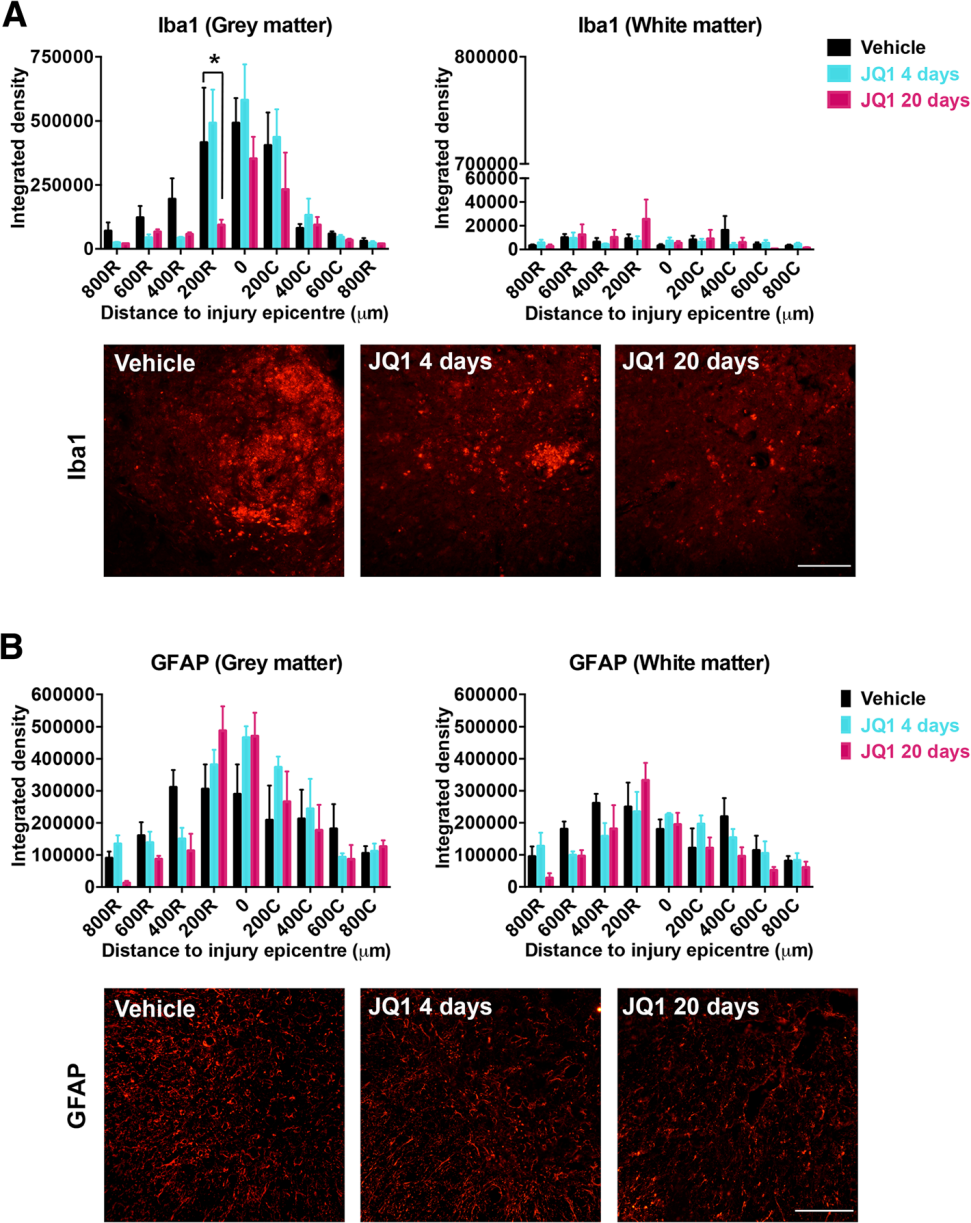
JQ1 treatment reduces macrophage and microglia reactivity after SCI. **a** Iba1 and **b** GFAP immunoreactivity quantification at 800 μm rostrally and caudally from the injury epicenter. Histograms represent the mean integrated density ± SEM quantified in gray (left) and white (right) matter in the ventral zone of the spinal cord sections. Representative images of Iba1 (**a**) and GFAP (**b**) at 200 and 400 μm, respectively, rostral to the epicenter are shown. Scale bar = 100 μm. *N* = 4–5 mice per group. *p < 0.05 compared to vehicle-treated animals and ^#^*p* < 0.05 compared to short-term-treated animals as calculated by two-way ANOVA with Tukey post hoc test

To further study the role of JQ1 in modulating the inflammatory response after SCI, we evaluated the expression of two hallmark markers of inflammation at longer time periods. Sham or SCI mice were treated with JQ1 or vehicle during 4 or 20 days, and immunoreactivity of microglia/macrophages and astrocytes was analyzed at 28 days post-injury. Iba1 staining detects both quiescent and reactive microglia and also infiltrated macrophages. The long-term JQ1 treatment showed a reduced immunoreactivity of Iba1 at the 200 μm rostrally to the lesion site (Fig. 3a), compared to vehicle treatment. The labeling for GFAP revealed that JQ1 did not affect astroglial reactivity (Fig. 3b). These results are consistent with our previous RT-qPCR findings at 72 h, showing no significant differences in mRNA expression of GFAP after JQ1 administration (Additional file 1: Figure S1B). Therefore, these results confirm that BET inhibition reduces microglial reactivity without affecting astroglial reactivity.

### BET inhibition promotes tissue sparing and neuronal preservation after SCI

**Fig. 4:**
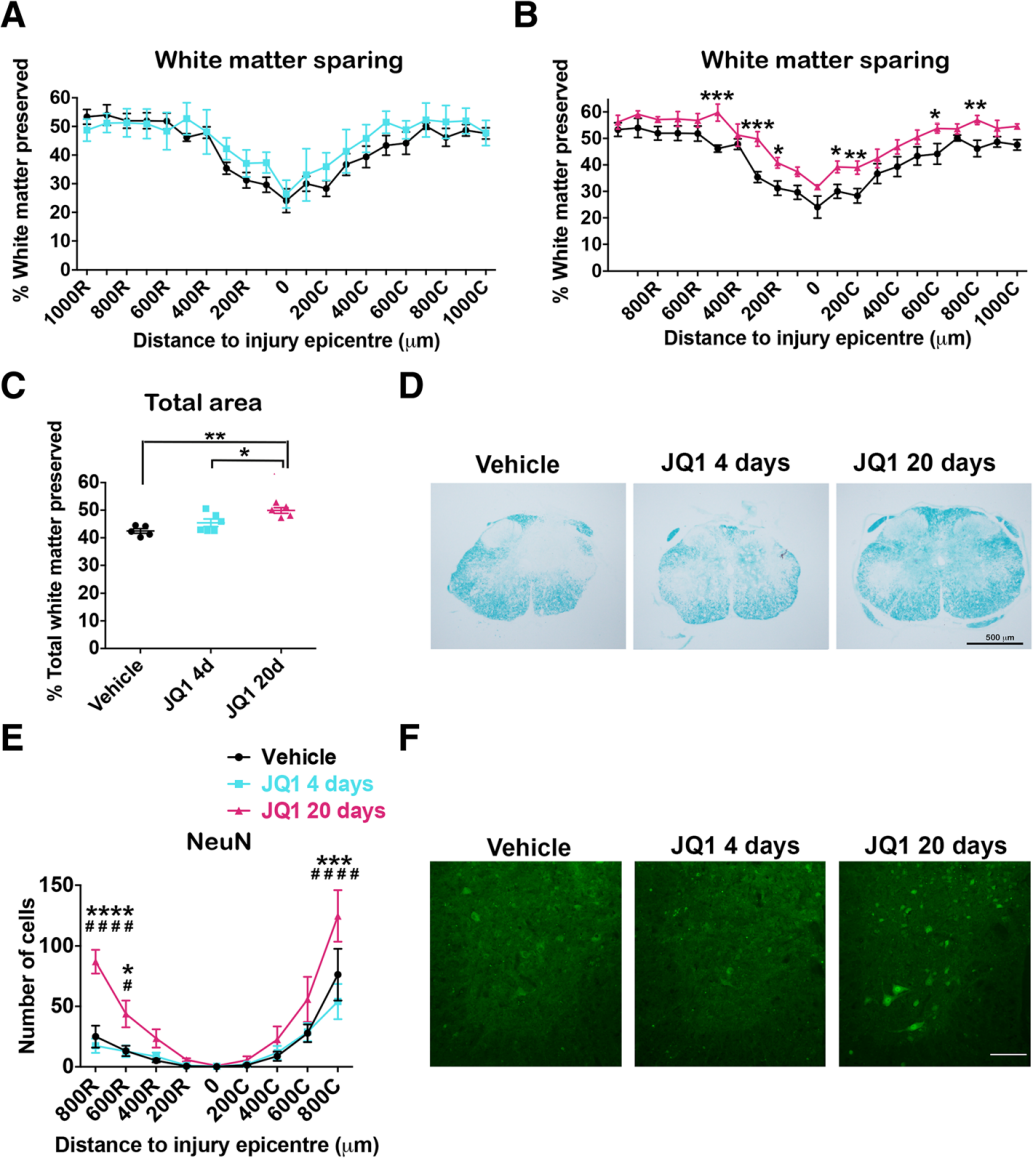
BET inhibition increases tissue preservation and neuronal survival after SCI. **a**, **b** Quantification of the percentage of white matter sparing, with respect to the total area of the spinal cord, from the injury epicenter up to 1000 μm rostral and caudally. **c** Percentage of total spared white matter in 2-mm spinal cord samples from different mouse groups. **d** Representative micrographs of sections stained with LFB at 300 μm rostral to the epicenter from vehicle-treated, JQ1 short-term-treated and JQ1 long-term-treated animals. **e** Quantification of ventral horn neuron survival from the injury epicenter to 800 μm rostral and caudally. **f** Representative micrographs showing sparing ventral horn neurons from vehicle-treated, JQ1 short-term-treated and JQ1 long-term-treated animals at 600 μm rostral to the lesion site. *N* = 4–5 mice per group. Scale bars, 500 μm (**d**, **f**). *p < 0.05, ***p < 0.005, and ****p < 0.001 compared to vehicle-treated group and ^#^*p* < 0.05 and ^####^*p* < 0.001 compared to short-term-treated group as calculated by two-way ANOVA with Tukey post hoc test. Data are represented as mean ± SEM

The LFB staining results revealed that administration of JQ1 for 20 days led to significant white matter sparing in rostral and caudal areas to the injury epicenter compared to vehicle-treated mice (Fig. 4b). Besides, the total labeled myelin area was significantly higher in long-term JQ1-treated mice than in vehicle-treated mice (Fig. 4c).

The number of surviving neurons in the ventral horn was counted at the epicenter of the injury and at 200, 400, 600, and 800 μm rostral and caudally. While quantitative analysis of NeuN staining showed that most neurons died at the injury site and surrounding areas in the three groups of mice, neuron survival was significantly increased at different distances from the epicenter. As it is shown in Fig. 4e, f, neuronal survival was significantly increased at 600 and 800 μm rostral from the epicenter, as well as at 800 μm caudal in long-term JQ1-treated animals compared to vehicle- and to short-term-treated mice (Fig. 4e, f). Overall, histopathological analysis revealed that long-term treatment of JQ1 conferred significant neuroprotection after SCI.

### BET inhibition enhances functional recovery after SCI

**Fig. 5:**
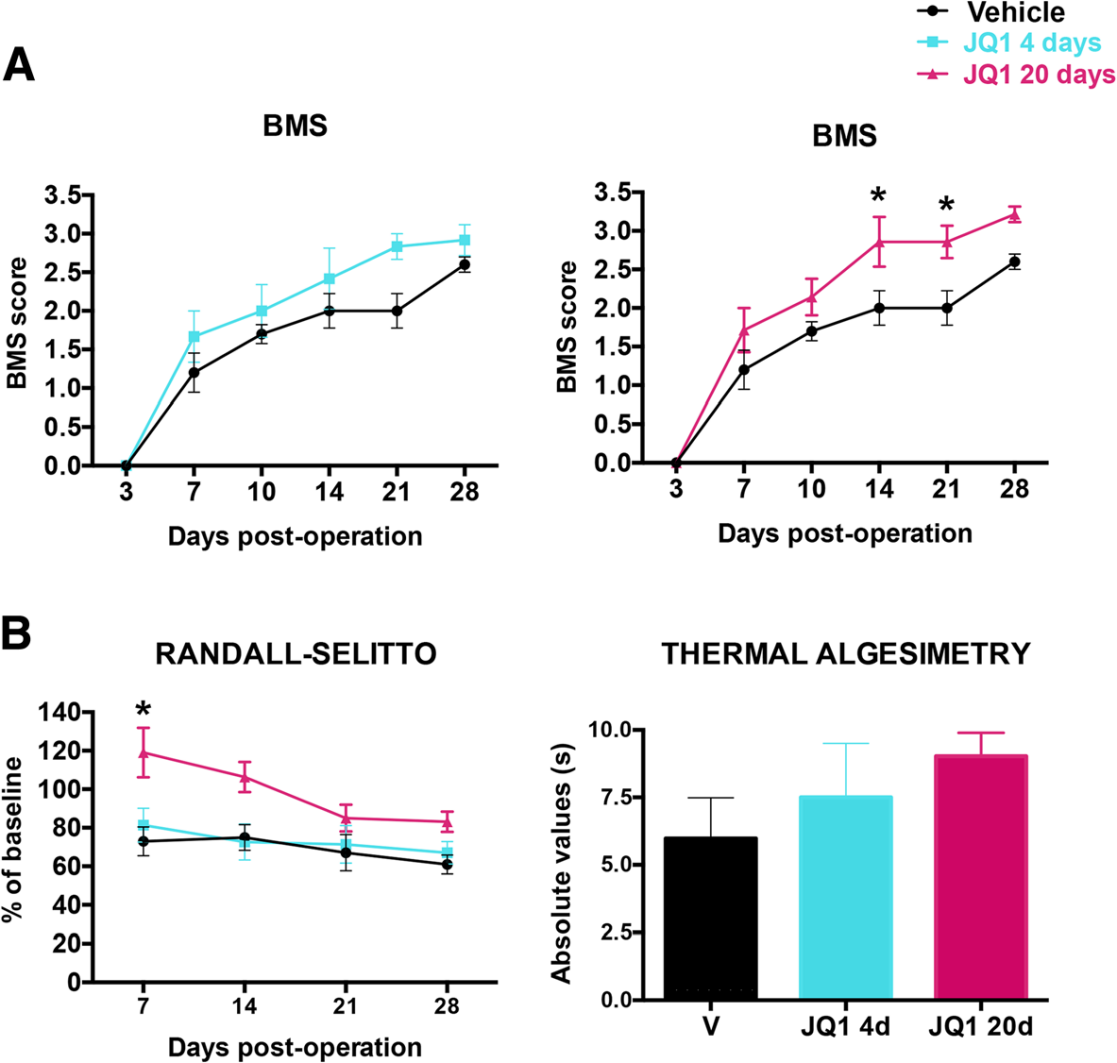
BET inhibition treatment increases functional outcome and reduces neuropathic pain after SCI. **a** BMS locomotion score along 28 days post-contusion. **b** Mechanical algesimetry results (left), expressed as percentage of baseline values, during follow-up, and thermal algesimetry results (right), expressed as absolute values in seconds, recorded at 28 days post-operation. *N* = 5–6 mice per group; *p < 0.05 as calculated by two-way ANOVA followed by Sidak correction for the multiple comparisons. Data are represented as mean ± SEM

To assess whether BET inhibition had an effect on functional recovery after SCI, locomotion was tested in an open field and scored with BMS scale. As shown in Fig. 5a, a gradual recovery of hindlimb locomotion was observed over the weeks following SCI in all mice. However, only mice that received JQ1 long-term treatment (*n* = 5) showed significant improvement of BMS scores at 14 and 21 days compared to vehicle-treated animals. Despite JQ1 administration during the first 4 days after SCI (*n* = 6) slightly increased the BMS score, this increase was not significantly different from vehicle-treated mice at any time point.

Finally, neuropathic pain was evaluated with Randall-Selitto and thermal algesimetry tests (Fig. 5b). Mice treated with JQ1 for 20 days presented reduced mechanical allodynia compared to the other groups, with significant differences at 7 days. The thermal algesimetry test showed a non-significant increase in the withdrawal threshold to hot stimulation in JQ1-treated mice.

Thus, BET inhibition enhances functional outcome and has a transient effect on neuropathic pain after SCI.

## Discussion

Although the interest in epigenetics has rapidly increased over the last years, the function of epigenetic signaling in central nervous system trauma remains largely unexplored. Thus, the overall goal of this study was to investigate the therapeutic role of targeting the epigenome, specifically BET proteins, after SCI. For this purpose, we used in vitro and in vivo approaches to study the effect of the BET inhibitor JQ1 on inflammation, neuroprotection, functional recovery, and ultimately on neuropathic pain. The results of the present study indicate that inhibition of BET proteins reduces inflammation and may represent a novel therapeutic target for SCI. We have observed that JQ1 decreased pro-inflammatory and increased anti-inflammatory cytokine expression acutely after SCI. We used JQ1 treatment at two different time regimes and observed that the prolonged treatment with JQ1 reduced the inflammatory response and increased neuroprotection. Altogether, these neuroimmune outcomes were associated with an improvement of functional recovery and reduction in neuropathic pain. Overall, the results of the present study highlight the importance of epigenetic regulation on inflammation after SCI.

### Effects of BET inhibition on the inflammatory response

One of the major findings of the present study is that BET inhibition, besides reducing pro-inflammatory cytokine expression, which had been previously described [15, 21], enhances anti-inflammatory cytokine production. This effect was observed after SCI and in a primary culture of bone marrow-derived macrophages. BET proteins are epigenetic readers, i.e., they read the acetylated residues in histones located in specific promoters, and promote gene expression. Thus, its inhibition commonly reduces gene expression. The reduction of these inflammatory-related genes, which are mediated by NF-kB signaling, can be explained by the disruption of the normal functioning of BET proteins by JQ1 treatment during an inflammatory response [22, 23]. However, molecular mechanisms for increasing anti-inflammatory cytokine production after BET inhibition are elusive. This effect may be indirect. However, BET protein BRD4 can confer transcriptional repression by interacting with several repressive complexes [24–26]. Thus, it might be possible that BET inhibition treatment produces chromatin release of these complexes and triggers promoter de-repression as suggested previously [25], inducing increases of anti-inflammatory cytokine production. However, further experiments should be performed to decipher the molecular events undergoing these effects.

The importance of macrophages and microglia in SCI lies in their influence to exacerbate inflammation as well as repair. These opposite actions are possible through macrophage/microglia polarization [5]. The raised levels of the anti-inflammatory cytokines IL-4, IL-10, and IL-13 observed after JQ1 treatment are particularly interesting since many studies have highlighted their relevance in inducing an alternative activation to macrophages, which is linked to SCI recovery [1, 27, 28]. However, we found that the enhancement in anti-inflammatory cytokines could reduce the expression of the M1 marker iNOS but could not increase M2 markers (ARG1 and CD206), and thus, it might not affect the switch of macrophages into an anti-inflammatory phenotype. Accordingly to literature, this result could be explained because not all of the M1 and M2 markers seem to change expression in a coordinated fashion after SCI [5]. Nevertheless, a higher number of M1/M2 markers and fluorescence-activated cell sorting (FACS) of macrophages and microglia harvested from the injured spinal cord would provide a better understanding of the changes in macrophage phenotype after JQ1. Besides, it might be possible also that the dosage or timing of the treatment was not sufficient to induce a substantial increase of IL-4 to observe these effects. In fact, we were not able to detect IL-4 protein with the cytokine protein expression assay (data not shown). We cannot affirm that BET inhibition did not enhance IL-4 production, since the detection range of the assay was higher than the protein biological levels. However, these levels are below those needed to produce a shift towards an M2 phenotype.

### Effects of BET inhibition on neuroprotection and functional recovery

A challenge for researchers who work in the CNS trauma field is to control the cross-talk between the immune and nervous system to prevent neurodegeneration and tissue loss. In the current study, we observed that JQ1 can promote neuroprotection, tissue preservation, and locomotion improvement. These beneficial effects may be attributed to the role of JQ1 modulating inflammation. The observed reduction in pro-inflammatory cytokines and also the increase of anti-inflammatory cytokines may overcome the neurotoxic environment of the acute phase of SCI, which eventually culminates in neuronal and oligodendrocyte death as well as tissue loss. In the end, these beneficial outcomes might improve functional recovery.

This conclusion is formed in view of the substantial literature related to the neurotoxic effects of TNF-α and IL-1β and the neuroprotective effects of IL-4 and IL-10 [5, 29]. Regarding pro-inflammatory cytokines, it has been described that blocking TNF-α and IL-1β confers neuroprotection in SCI and stroke models [8]. For instance, it has been reported that the genetic or pharmacological inhibition of TNF-α reduces inflammation, injured tissue, and the number of apoptotic cells which overall ameliorate the recovery of limb function [30]. Considering anti-inflammatory cytokines, it has been demonstrated that a treatment of IL-4 reduces tissue damage and locomotion impairment after SCI [28].

Despite our LFB staining revealed an enhanced tissue preservation, this fact cannot be directly linked to a protective action against oligodendrocyte death. Thus, future histological procedures will be performed to analyze the effects of JQ1 treatment on oligodendrocytes. However, we could observe enhanced neuronal survival, demonstrating a neuroprotective effect of JQ1 on these cells after SCI, probably due by the decreased cytotoxic environment produced by JQ1 treatment. This preservation was statistically significant in areas adjacent to the injury epicenter.

Our studies demonstrate an acute effect of BET inhibition by reducing pro-inflammatory and increasing anti-inflammatory cytokine/chemokine levels after SCI. However, neuroprotection was observed only after a prolonged treatment during 20 days and not after a short treatment of 4 days, timing when most of the enhanced cytokines/chemokines return to control levels after SCI [8]. Thus, other chronic effects of BET inhibition may also account for these mechanisms. It may be possible that a sustained increase of anti-inflammatory cytokines could promote prolonged neuroprotective/regenerative effects. In fact, IL-4, IL-10, and IL-13 high levels promote enhanced axonal regeneration [31, 32], promote neuroprotection after SCI [29, 33], and reduce demyelination in multiple sclerosis [34]. Besides, it is reported that BET proteins play a role in most of neuronal types [21, 35]. Therefore, further studies should be performed analyzing the role of BET inhibition in neuroprotection, axonal regeneration, and demyelination after a prolonged treatment.

### Effects of BET inhibition on neuropathic pain

Functional assessment of neuropathic pain showed preservation of the mechanical threshold at 7 days post-injury. There are 2 main mechanisms thought to contribute to hyperexcitability and therefore to the ontogeny of neuropathic pain (reviewed in [36]). One is the release of neuromodulators such as pro-inflammatory cytokines and chemokines, which enhance neuronal excitability. The other one is the altered expression, trafficking, and functioning of receptors and ion channels expressed by primary sensory neurons. Thus, probably BET treatment is only effective on reducing pro-inflammatory mediators, such as TNF-α, CCL2, and CX3CR1, which are directly implicated in neuropathic pain. Of interest, both CCR2 and CX3CR1-knockout mice exhibit reduced neuropathic pain [37]. A combination with any other epigenetic treatment that regulates gene expression in primary sensory neurons could produce a prolonged effect on neuropathic pain.

We and several authors report beneficial effects of BET inhibition reducing tumor growth [38–40], increasing brain plasticity [41], promoting neuroprotection [42], and decreasing inflammation in several pathologies [43, 44]. However, higher doses of JQ1 can block memory formation [35] or produce lymphoid and hematopoietic toxicity in mice [45] and can even enhance undesirable virus replication [46, 47]. In fact, we observed that doubling the dose of the compound produced a decrease in body weight and mouse death after SCI (data not shown). Therefore, further studies carefully dissecting the dosage, analyzing the molecular mechanisms of action, and determining undesirable effects of BET inhibitors should be performed to find the balance between beneficial and detrimental effects of BET inhibition in each pathological condition and be able to progress towards clinical trials [48].

## Conclusions

This work elucidates an epigenetic mechanism which contributes to the pathogenesis of inflammation and SCI. Although BET inhibition has been previously reported to have an anti-inflammatory effect after SCI [21], a beneficial outcome of BET inhibition treatment after SCI has never been reported before. Thus, the present study demonstrates for the first time that BET proteins are a target for the treatment of SCI, promoting neuroprotection, behavioral recovery, and decreased neuropathic pain. Neuroinflammation is a common feature of several human neurodegenerative diseases. Therefore, the discoveries from the present project may provide therapeutic innovation for many other pathologies, increasing the translational impact of the present study.

## Additional files

Additional file 1: JQ1 treatment modifies the M1 marker INOS but do not modify markers of M2 macrophage phenotype, or astrocyte reactivity. Real-time PCR quantification of (A) M1 and M2 markers and (B) macrophage and glial markers at 4 and 72 h after SCI, normalized to the GAPDH levels. *N* =3-4 mice/group. *p < 0.05, ***p < 0.005, as calculated by one-way ANOVA followed by Tukey post-hoc test. Data are represented as mean ± SEM fold changes of gene expression. (TIF 1771 kb)

Additional file 2: BET inhibition affects macrophage reactivity in vitro. Real-time PCR quantification of (A) pro-inflammatory and (B) anti-inflammatory cytokines at 3 h after LPS (100 ng/ml) stimulation, normalized to the GAPDH levels. Experiments were repeated 3 independent times. *p < 0.05, **p < 0.01, ***p< 0.005, as calculated by one-way ANOVA followed by Tukey post-hoc test. Data are represented as mean ± SEM fold changes of gene expression. (TIF 1378 kb)

## Data Availability

The datasets used and/or analyzed during the current study are available from the corresponding author on reasonable request.

## Abbreviations

ARG1: Arginase 1
BET: Bromodomain and extra-terminal domain
BMS: Basso Mouse Scale
CCL: C-C motif chemokine ligand
CD206: Cluster of differentiation 2006
CXCR: C-X-C chemokine receptor
FACS: Fluorescence-activated cell sorting
GFAP: Glial fibrillary acidic protein
Iba1: Ionized calcium-binding adaptor molecule 1
IL: Interleukin
LBF: Luxol Fast Blue
LPS: Lipopolysaccharide
NF-kB: Nuclear factor kappa-light-chain-enhancer of activated B cells
SCI: Spinal cord injury
TNF-α: Tumor necrosis factor-α
