# Correction to: BET protein inhibition regulates cytokine production and promotes neuroprotection after spinal cord injury

**DOI:** 10.1186/s12974-022-02590-z

**Published:** 2022-09-19

**Authors:** Judith Sánchez-Ventura, Jesús Amo-Aparicio, Xavier Navarro, Clara Penas

**Affiliations:** Institut of Neurosciences, Dept Cell Biology, Physiology and Immunology, Centro de Investigación Biomédica en Red sobre Enfermedades Neurodegenerativas (CIBERNED), Universitat Autonoma de Barcelona, Barcelona, Spain

## Correction to:Journal of Neuroinflammation (2019) 16:124 https://doi.org/10.1186/s12974-019-1511-7

Following publication of the original article [1], the authors identified an error in Table 1.

The problem is that 2 sequences of the primer list in Table 1 were wrong. In red are the ones that should be changed. The corrected version of Table 1 is given.

**Table 1:**
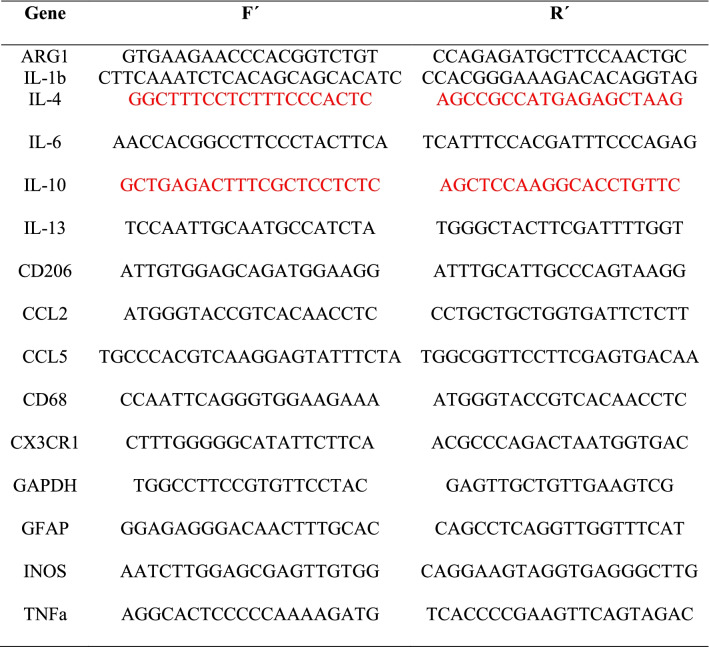
List of primers used in RT-qPCR
