# Supplementary figures and images for: BET protein inhibition regulates cytokine production and promotes neuroprotection after spinal cord injury
Source: J Neuroinflammation. 2019 Jun 11;16:124. doi: 10.1186/s12974-019-1511-7 (PMC6560758; doi:10.1186/s12974-019-1511-7)

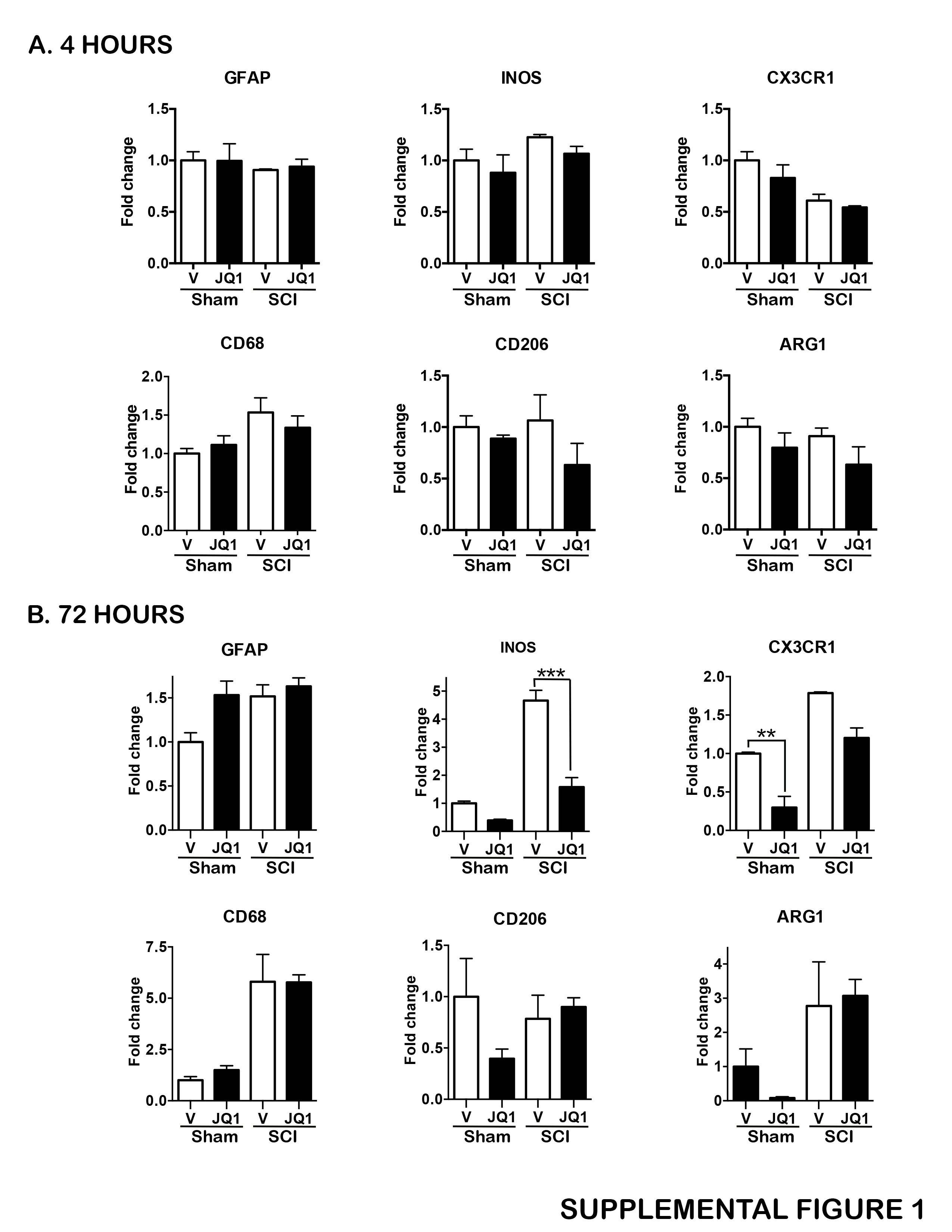

Supplement: Supplementary file 1 — JQ1 treatment modifies the M1 marker INOS but do not modify markers of M2 macrophage phenotype, or astrocyte reactivity. Real-time PCR quantification of (A) M1 and M2 markers and (B) macrophage and glial markers at 4 and 72 h after SCI, normalized to the GAPDH levels. N =3-4 mice/group. *p < 0.05, ***p < 0.005, as calculated by one-way ANOVA followed by Tukey post-hoc test. Data are represented as mean ± SEM fold changes of gene expression. (TIF 1771 kb) [file 12974_2019_1511_MOESM1_ESM.tif]

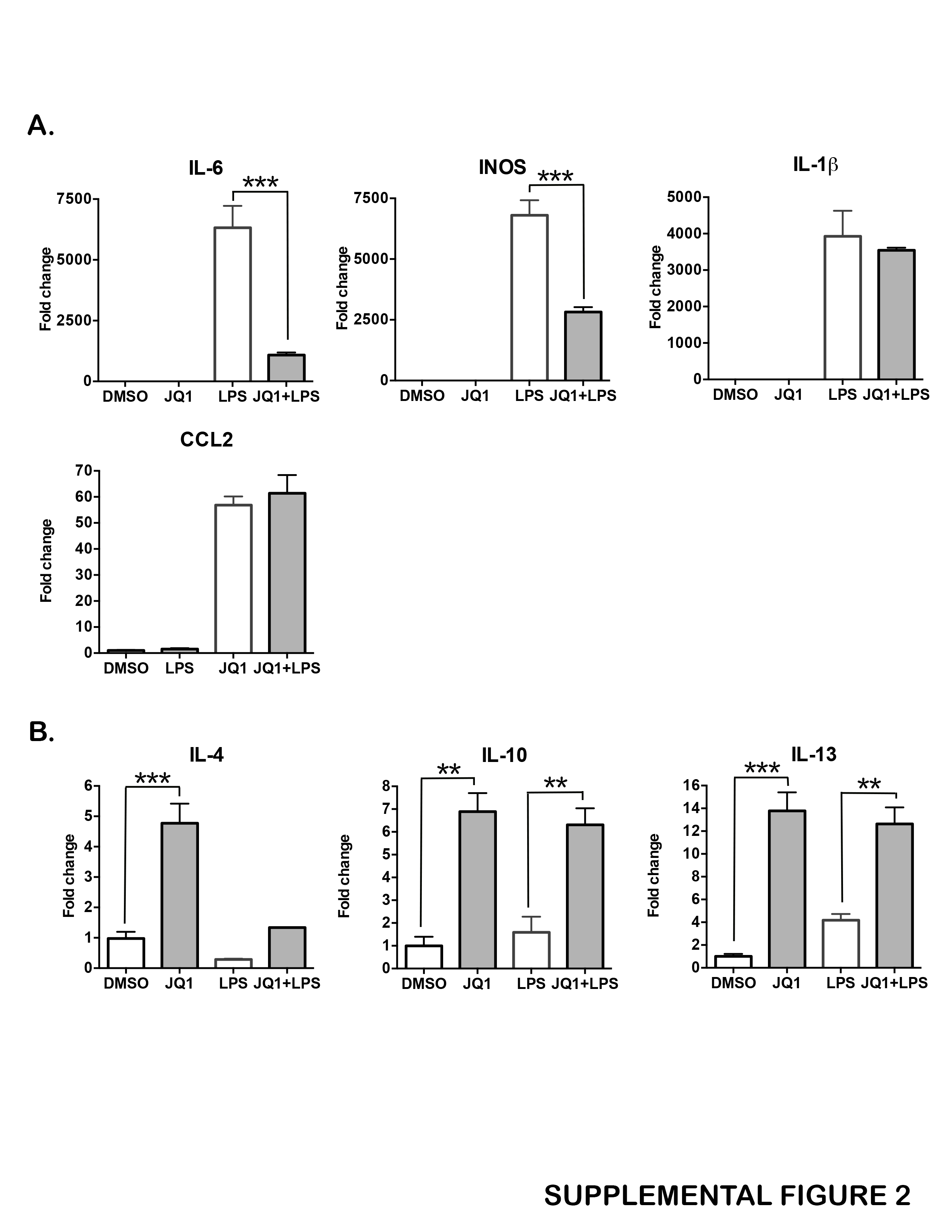

Supplement: Supplementary file 2 — BET inhibition affects macrophage reactivity in vitro. Real-time PCR quantification of (A) pro-inflammatory and (B) anti-inflammatory cytokines at 3 h after LPS (100 ng/ml) stimulation, normalized to the GAPDH levels. Experiments were repeated 3 independent times. *p < 0.05, **p < 0.01, ***p< 0.005, as calculated by one-way ANOVA followed by Tukey post-hoc test. Data are represented as mean ± SEM fold changes of gene expression. (TIF 1378 kb) [file 12974_2019_1511_MOESM2_ESM.tif]
